# Transcriptome Analysis of Adipose Tissue Indicates That the cAMP Signaling Pathway Affects the Feed Efficiency of Pigs

**DOI:** 10.3390/genes9070336

**Published:** 2018-07-04

**Authors:** Yueyuan Xu, Xiaolong Qi, Mingyang Hu, Ruiyi Lin, Ye Hou, Zhangxu Wang, Huanhuan Zhou, Yunxia Zhao, Yu Luan, Shuhong Zhao, Xinyun Li

**Affiliations:** 1 Key Laboratory of Agricultural Animal Genetics, Breeding, and Reproduction of the Ministry of Education and Key Laboratory of Swine Genetics and Breeding of Ministry of Agriculture, Huazhong Agricultural University, Wuhan 430070, China; yyxu@webmail.hzau.edu.cn (Y.X.); 15839752008@163.com (X.Q.); myhu@webmail.hzau.edu.cn (M.H); ruiyil@126.com (R.L.); houye2009@126.com (Y.H.); wzx66999@163.com (Z.W.); zhouhuanhuanst@163.com (H.Z.); zhaoyunxia126@126.com (Y.Z.); ly178574378@hotmail.com (Y.L.); shzhao@mail.hzau.edu.cn (S.Z.); 2 The Cooperative Innovation Center for Sustainable Pig Production, Wuhan 430070, China

**Keywords:** feed efficiency, adipose, cAMP, lipid metabolism, pig

## Abstract

Feed efficiency (FE) is one of the main factors that determine the production costs in the pig industry. In this study, RNA Sequencing (RNA-seq) was applied to identify genes and long intergenic non-coding RNAs (lincRNAs) that are differentially expressed (DE) in the adipose tissues of Yorkshire pigs with extremely high and low FE. In total, 147 annotated genes and 18 lincRNAs were identified as DE between high- and low-FE pigs. Seventeen DE lincRNAs were significantly correlated with 112 DE annotated genes at the transcriptional level. Gene ontology (GO) analysis revealed that DE genes were significantly associated with cyclic adenosine monophosphate (cAMP) metabolic process and Ca^2+^ binding. cAMP, a second messenger has an important role in lipolysis, and its expression is influenced by Ca^2+^ levels. In high-FE pigs, nine DE genes with Ca^2+^ binding function, were down-regulated, whereas *S100G*, which encodes calbindin D9K that serve as a Ca^2+^ bumper, was up-regulated. Furthermore, *ATP2B2*, *ATP1A4*, and *VIPR2*, which participate in the cAMP signaling pathway, were down-regulated in the upstream of lipolysis pathways. In high-FE pigs, the key genes involved in the lipid biosynthetic process (*ELOVL7* and *B4GALT6*), fatty acid oxidation (*ABCD2* and *NR4A3*), and lipid homeostasis (*C1QTNF3* and *ABCB4*) were down-regulated. These results suggested that cAMP was involved in the regulation on FE of pigs by affecting lipid metabolism in adipose tissues.

## 1. Introduction

Feed accounts for approximately 60–70% of the total cost of pork production [1]. Increasing the feed efficiency (FE) may effectively reduce production costs in the swine industry. Residual feed intake (RFI) and feed conversion ratio (FCR) are two main measures of FE. RFI is the difference between the actual feed intake of pigs and the predicted feed intake required for maintaining and gaining body weight, and FCR is defined as the ratio of feed intake to the daily weight gain of pigs [2]. Low FCR and RFI values correspond to high FE [2].

Previous studies have applied microarray data analyses to identify FE-associated genes in porcine adipose tissue [3,4,5]. The lipid metabolic process was reported to be enriched by down-regulated genes of adipose tissues in high-FE pigs compared to low-FE pigs [3]. The top differentially expressed (DE) genes are involved in lipid catabolic process and are expressed in adipose tissues of high-FE pigs at levels 5-fold higher than those in adipose tissues of low-FE pigs [4]. Likewise, genes related to lipid catabolism are up-regulated in adipose tissues of high-FE pigs relative to that in adipose tissues of low-FE pigs [5]. These results indicate that lipid metabolism in adipose tissue plays an important role in regulating the FE of pigs.

Ca^2+^ and cyclic adenosine monophosphate (cAMP) are the two most common second messengers in eukaryotic cells [6]. The intracellular regulation of lipolysis in human adipose tissue is affected by changes in free Ca^2+^ and cAMP levels [7]. In human adipose-derived stem cells, high intracellular Ca^2+^ levels can restrain adipogenesis [8]. Moreover, in human adipocytes, high Ca^2+^ levels exert an antilipolytic effect mainly through phosphodiesterase (PDE) activation, which reduces cAMP and hormone-sensitive lipase (HSL) phosphorylation, and consequently inhibits lipolysis [9]. The results of trials on mice also showed that cAMP-dependent protein kinase A (PKA) pathway leads to lipolysis by regulating phosphorylation levels of HSL [10,11]. Furthermore, lipolysis can be induced by agonists that couple to adenylate cyclase via the G-protein subunit Gs; this coupling activates the cAMP-mediated PKA pathway in adipocytes [12]. In pig adipose tissues, cAMP phosphodiesterase as an intermediation, influences the lipolysis process [13]; furthermore, a possible link between obesity and cAMP pathway was demonstrated by feeding treatment trails [14]. Therefore, Ca^2+^ and cAMP are closely related to lipid metabolism.

Long noncoding RNAs (lncRNAs) are a category of transcripts, longer than 200 nucleotides (nt), that do not encode any proteins. According to position relative to protein-coding genes, lncRNAs can be divided into three classes: antisense transcripts, long intronic noncoding RNAs, and long intergenic noncoding RNAs (lincRNAs) [15]. LincRNAs are associated with adipogenesis and lipid metabolism. For example, several lincRNAs that are functionally required for proper adipogenesis have been identified in the adipose tissues of mice [16]. Numerous lincRNAs that are DE in the adipose tissues of different pig breeds are enriched in the processes and pathways of fat-cell differentiation and fatty acid metabolism [17]. In mice, the lincRNAs *MALAT1*, *H19*, and *lncHR1* regulate hepatic lipogenesis by inducing sterol regulatory element-binding protein (SREBP)-1c protein expression, which enhances the transcription of genes required for fatty acid synthesis [18,19,20]. We previously identified several FE-related lincRNAs in porcine hepatic tissue [21]. However, FE-related lincRNAs that are DE in porcine adipose tissue, remain unknown.

Here, we applied RNA sequencing analysis to identify a catalogue of DE genes and lincRNAs between the adipose tissues of pigs with high and low FE. Gene ontology (GO) and Kyoto Encyclopedia of Genes and Genomes (KEGG) pathway analysis revealed that the cAMP signal affects the FE of pigs by mediating lipid metabolism. Thus, the molecular mechanism that underlies the regulatory effect of cAMP on the FE of pigs warrants further exploration.

## 2. Materials and Methods

### 2.1. Animals and Sample Collection

The animal source was the same as our previous works, feed intake of 236 Yorkshire pigs were measured using ACEMA 64 (Pontivy, France) automated individual feeding systems under the same conditions [21,22]. Three pigs with the highest FE (high-FE) and three pigs with the lowest FE (low-FE) were selected based on their RFI values which were significantly different (*p* < 0.05), but their total weight gain and feed time were not different [22]. Adipose tissue samples were collected from pigs after slaughter, immediately snap-frozen in liquid nitrogen, and stored at −80 °C until used in RNA extraction. Total RNA was isolated from the fragmented frozen adipose samples by using TRIzol reagent (Invitrogen, Carlsbad, CA, USA). All experimental protocols were approved in 2013 by the Ethics Committee of Huazhong Agricultural University (HZAUMU2013-0005).

### 2.2. Library Preparation and Sequencing

The total RNA from each tissue sample was used to RNA Sequencing (RNA-seq) library preparation with the TruSeq Total RNA Sample Preparation kit (Illumina, San Diego, CA, USA). After quality control, six libraries on the same lane were sequenced using Illumina HiSeq high-throughput sequencing instrument with 150 PE reads. The clean data were obtained after cutting adapters and filtering reads with low average quality.

### 2.3. RNA Sequencing Analysis

Tophat (v2.1.1) [23] was used to align sequencing reads from each sample to the reference genome of *Sus scrofa* v. 10.2 and 11.1. The reference genome and annotation file were downloaded from Ensembl (http://www.ensembl.org/info/data/ftp/index.html). Mapped reads in the intergenic region were also compared with annotated lincRNAs for alignment with the *S. scrofa* 10.2 genome [24]. HTSeq-count [25] was used to count the reads in lincRNAs and protein-coding genes.

### 2.4. Differential Expression Analysis and Real-Time Quantitative PCR Validation

DEseq2 [26] was used to identify DE genes and lincRNAs between high- and low-FE pigs. Annotated protein-coding genes and lincRNAs showing |log2FoldChange| (|log2FC|) ≥ 1 and *p*-value < 0.05 were considered to be DE.

The relative expression levels of DE genes in adipose tissues were quantified through a real-time quantitative PCR (RT-qPCR) detecting system. Total RNA was extracted for RNA sequencing as described. Complementary DNA (cDNA) fragments were obtained via reverse transcription by using a RevertAid First Strand cDNA Synthesis Kit (K1621, Thermo Scientific, Waltham, MA, USA). Oligonucleotide primers for three DE genes and three lincRNAs were designed with Primer7 software. Primer sequences are listed in Supplementary Table S1. qPCR was conducted with SYBR Green PCR MasterMix (Toyobo Co., Ltd., Osaka, Osaka Prefecture, Japan) on a Bio-Rad CFX384 Real-Time System (BioRad Laboratories, Inc., Hercules, CA, USA) in accordance with the manufacturer’s instruction. The 2^−ΔΔCt^ method was applied to normalize the relative expression levels of DE genes and lincRNAs to those of the *ACTB* gene, which is stably expressed in adipose tissues (Figure S1) [27]. The Student’s *t*-test was used to analyze the differential expression of genes between high- and low-FE pigs.

### 2.5. Correlation Analysis

The correlation analysis of the DE genes and lincRNAs in adipose tissues was performed using two methods, weighted correlation network analysis (WGCNA) [28] and Pearson correlation analysis. A weight co-expression network was constructed on the basis of the normalized count matrix from DEseq2 by using WGCNA package in the R environment. A power value of 7 was selected as the soft threshold to maximize fitness to the scale-free topology of the whole network. All genes and lincRNAs were clustered into distinct modules through hierarchical clustering followed by dynamic tree cutting. DE genes and lincRNAs were filtered from modules containing DE lincRNAs. The weight values between DE genes and lincRNAs were yielded by the same modules. In addition, the Pearson correlation coefficients between DE genes and lincRNAs were also calculated in the R environment. Specifically, the criteria for significant lincRNAs annotated gene pairs were *p* < 0.05 and |R| > 0.82 in this method.

### 2.6. Gene Ontology Enrichment and Pathway Analysis

The homologous human gene symbols of the identified DE genes were used in the GO enrichment and KEGG pathway analysis, which were both implemented in the DAVID Bioinformatics Resources 6.8 version [29] (https://david.ncifcrf.gov/summary.jsp). The lists of human gene symbols were submitted to the website. The biological process enrichment terms of DE genes were obtained from a new GO category (GO Direct), which provides GO mappings that have been directly annotated by the source database. The pathways that involved the DE genes were retrieved from the KEGG pathway database. Cut-off criteria of both them were *p* value < 0.05. DE lincRNAs that were expressed in association with DE genes were also integrated in pathways. Finally, the visualization analysis of the pathway was performed using an open source software—Cytoscape [30].

## 3. Results

### 3.1. Mapping and Annotation of RNA Sequencing Data

Three RNA-seq libraries were sequenced from the adipose tissues of high- and low-FE pig groups. After trimming adaptors and filtering reads with low average quality, RNA-seq analysis yielded 33.8–39.9 million PE reads for all six samples (Table 1 and Table 2). A total of 30.2–37.8 million reads were aligned to *S. scrofa* v. 10.2 and 11.1 version of the porcine genome, and approximately 74.5–91.4% reads were uniquely aligned in pairs (Table 1 and Table 2). The clean data of RNA-seq have been submitted to the NCBI Sequence Read Archive under adipose tissue part of series SRP149276.

**Figure 1:**
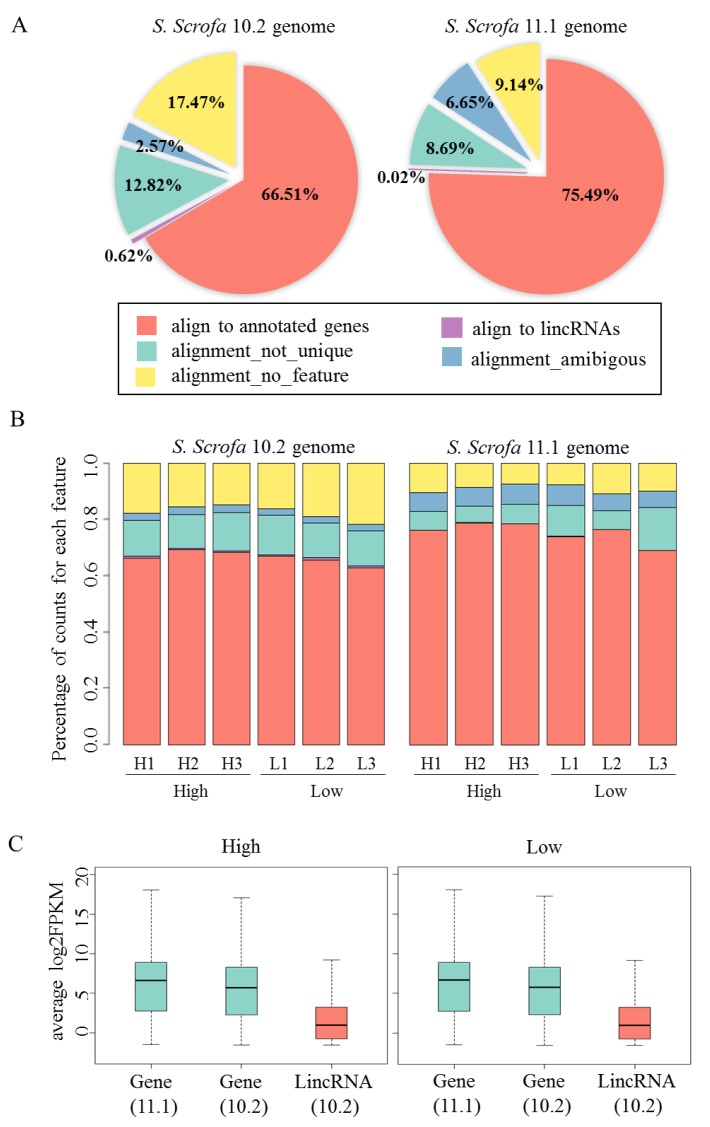
Annotation and alignment of RNA-seq read pairs from porcine adipose tissue with different genome version. (**A**) Distribution of average mapped read pairs of six samples on the *S scrofa* 10.2 (**left**) and *S. scrofa* 11.1 (**right**) genomes. In the pie charts, the percentages represent the mean of all six RNA-seq data. On average, over 66% unique read pairs were aligned to annotated genes. (**B**) Distribution of mapped read pairs in different samples. Samples that belong to the same genome group show similar distribution. The color of bar standing for the same meaning as pie charts. (**C**) Boxplots showing the expression patterns (scaled log2FPKM) of annotated genes and long intergenic non-coding RNAs (lincRNAs) in high Feed Efficiency (high-FE) (**left**) and low-FE (**right**) groups, respectively.

The majority of the mapped reads (66.51–75.49%) of all samples were uniquely distributed on the annotated genes. Meanwhile, the minority of mapped reads (9.14–17.47%) were not aligned to any feature. Moreover, ca. 0.62% of the mapped reads were located in the annotated lincRNA region of the *S. scrofa* 10.2 genome (Figure 1A). The mapped reads of each sample independently and similarly distributed in the same genomic region (Figure 1B). Comparison the high- and low- FE pig groups separately revealed that the expression levels of annotated genes were higher than that of lincRNAs. (Figure 1C). Although the alignment ratio of the mapped reads to the *S. scrofa* 11.1 genome was higher than that to the *S. scrofa* 10.2, the distribution of mapped reads and the expression level of transcripts of these two genome versions were similar.

### 3.2. DE Genes and lincRNAs between High- and Low-FE Pigs

**Figure 2:**
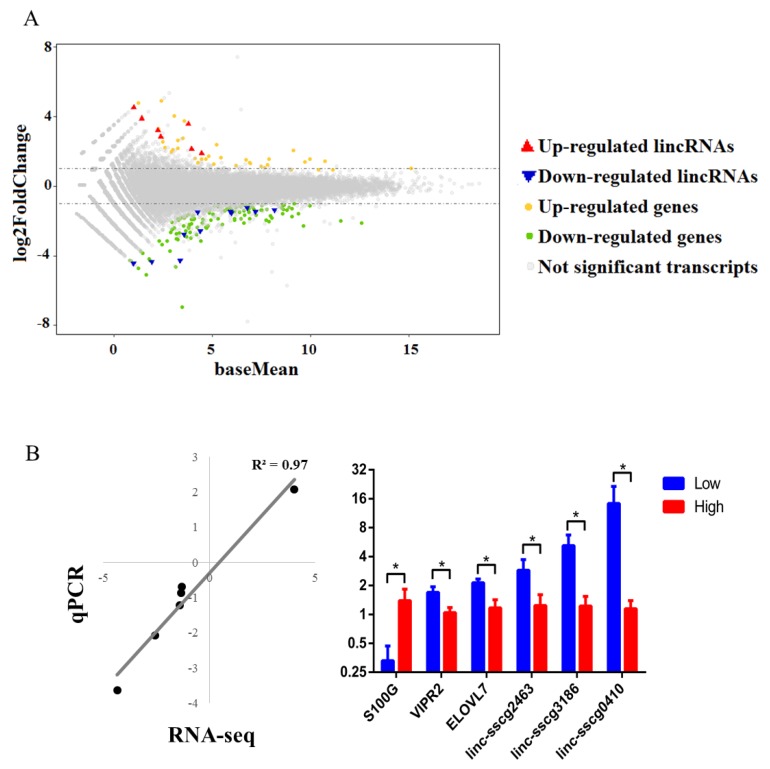
Differential expression analysis of annotated genes and lincRNAs between high- and low-FE pigs. (**A**) The distribution of fold changes in gene expression. Genes (lincRNAs) with absolute log2FoldChange > 1 are indicated in orange (red) and those with log2Foldchange < −1 are indicated in green (blue). (**B**) Quantitative-PCR (qPCR) analysis results of six selected DE genes and lincRNAs. Left: Scatter diagram showing the log2FC correlation of RNA-seq and qPCR. Right: Relative expression of selected DE genes and lincRNAs. Statistically significant differences between high- and low-FE pigs are indicated by * (*p* value < 0.05).

The *S. scrofa* 10.2 genome was used as a reference, given that it has been used to annotate lincRNAs from Zhou’s study [24]. A total of 147 genes and 18 lincRNAs were DE between high- and low-FE pigs (|log2FC| > 1, *p* < 0.05). Among these 147 annotated genes, 34 genes were up-regulated, and 113 genes were down-regulated in high-FE pigs (Figure 2A and File S1). Furthermore, 11 out of 18 annotated lincRNAs were down-regulated in high-FE pigs (Figure 2A and File S1).

RT- qPCR was employed to quantify the expression levels of DE genes and lincRNAs. Samples from six high-FE pigs and six low-FE pigs, which contained all samples for RNA-seq, were used for qPCR analysis. Here, six DE transcripts, including three DE genes and three DE lincRNAs from GO, pathway analysis and random selection, were selected for qPCR validation (Table S1). The qPCR results showed that all six selected DE transcripts were significantly DE between high- and low- pig groups. In addition, the log2FC values of qPCR and RNA-seq results were highly correlated (*R*^2^ = 0.97) (Figure 2B).

### 3.3. Correlation Analysis of DE Genes and lincRNAs

**Figure 3:**
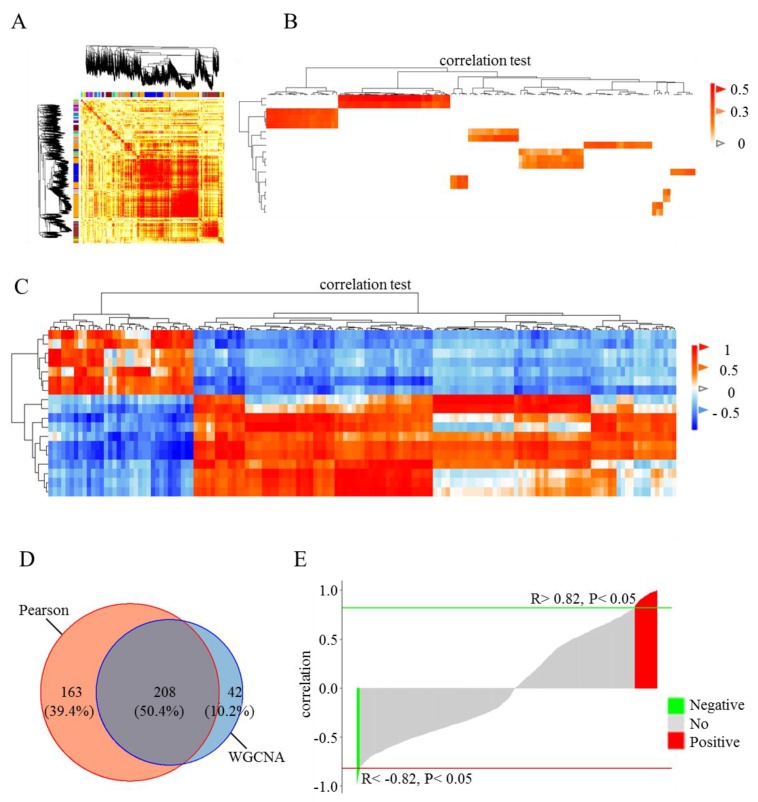
Correlation analysis of DE genes and lincRNAs in the adipose tissues of high- and low-FE pigs. Weighted correlation network analysis (WGCNA) was applied to identify modules of highly correlated factors, including genes and lincRNAs. (**A**) A total of 25 modules were identified on the basis of expression patterns, which are represented by the dendrogram and correlation heat map. (**B**) Correlations between differentially expressed (DE) genes and lincRNAs were identified through WGCNA. Dark color indicates higher correlation. Vertical and horizontal axes in the heat map represent lincRNAs and genes, respectively. (**C**) Correlations between DE genes and lincRNAs were estimated on the basis of the Pearson correlation coefficient. Lattices in red are highly positive, and those in blue are highly negative. (**D**) Venn diagram depicting the proportion of correlated lincRNA-gene pairs detected on the basis of WGCNA and Pearson correlation. (**E**) Bar plot showing the Pearson correlation coefficient of overlapping lincRNA-gene pairs. Red and green bar plots represent positively and negatively correlated pairs, respectively.

To explore the relationship between DE lincRNAs and FE, WGCNA and Pearson correlation analysis between DE genes and lincRNAs were performed (Figure 3). In WGCNA, all genes and lincRNAs were divided into 25 modules. All pairs of elements (genes and lincRNAs) in the same module exhibited higher co-expression than random pairs of elements from different modules (Figure 3A). On the basis of this result, nine modules that included DE lincRNAs were selected for the examination of the weight of lincRNA–gene pairs in the same modules. Therefore, 250 DE lincRNA–gene pairs were obtained. These DE lincRNAs-gene pairs included 119 DE annotated genes and 18 DE lincRNAs (Figure 3B and File S2). The Pearson correlation coefficient of the DE lincRNAs and genes was calculated. A total of 371 DE lincRNAs–gene pairs were significantly correlated and included 133 DE annotated genes and 18 DE lincRNAs (*p*-value < 0.05, |R| > 0.82), (Figure 3C and File S2). Overall, 208 correlated pairs of DE lincRNAs and genes were identified from both WGCNA and Pearson correlation analysis (Figure 3D and File S2). Among these 208 correlated pairs, 184 were positively correlated (Figure 3E and File S2).

### 3.4. GO Enrichment and Pathway Analysis 

**Figure 4:**
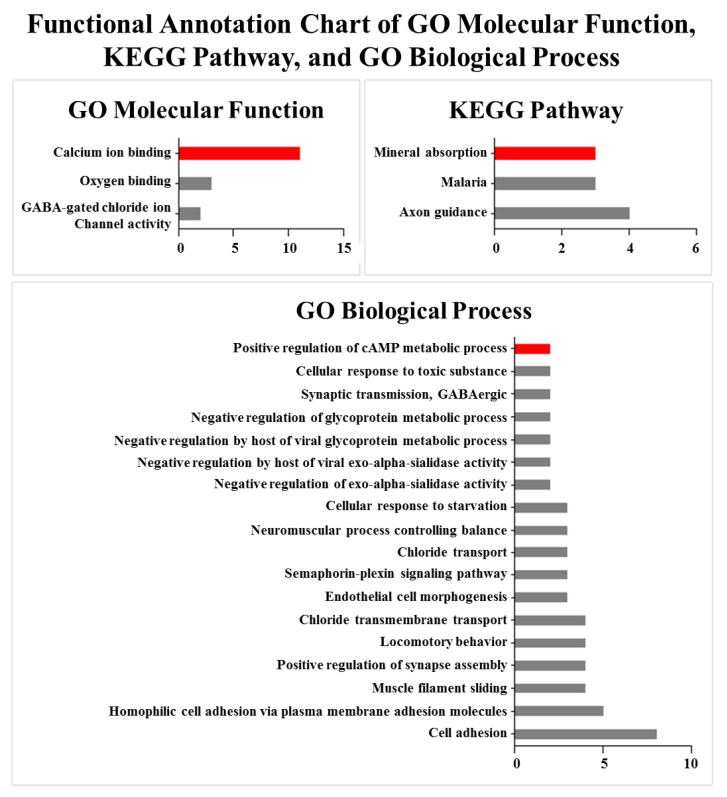
Results of functional enrichment analysis of DE genes between high- and low-FE pigs in genes ontology (GO) molecular function, Kyoto Encyclopedia of Genes and Genomes (KEGG) pathway, and GO biological process.

**Figure 5:**
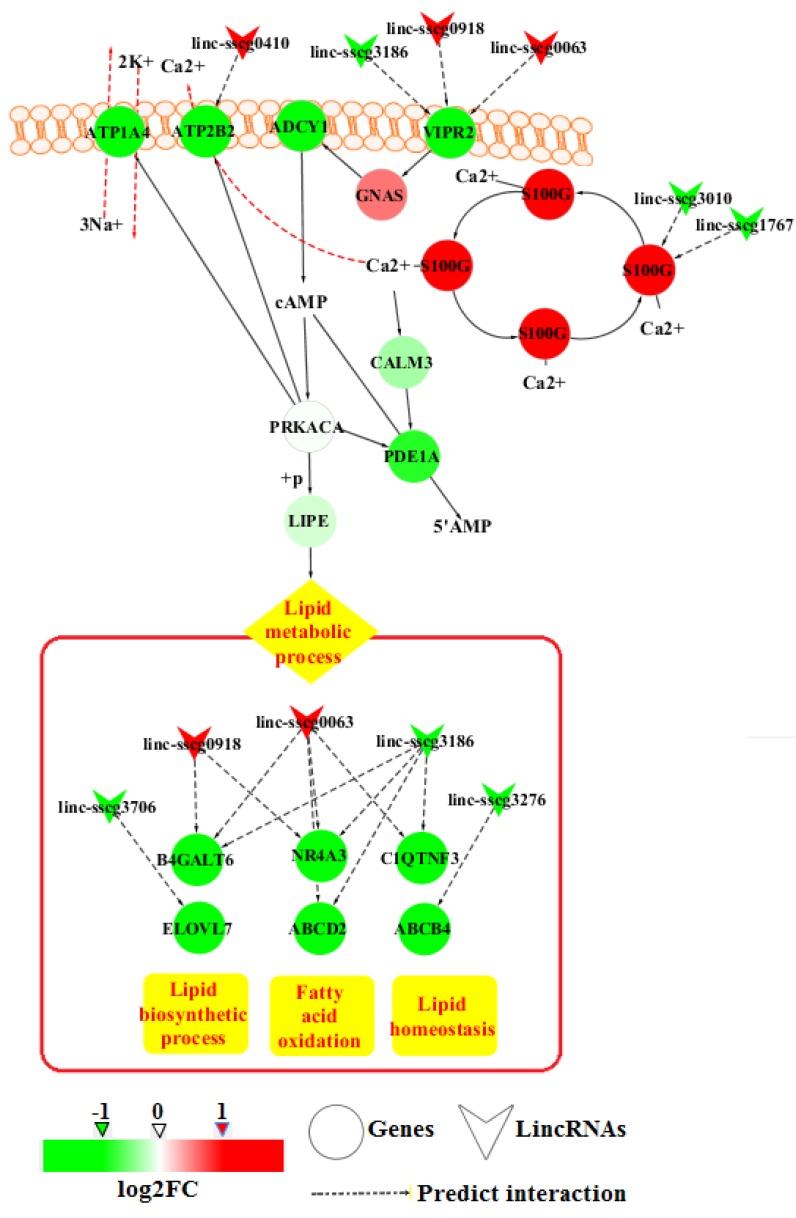
Potential pathways of the annotated DE genes and lincRNAs in the adipose tissues of high- and low-FE pigs. Red and pink colors indicate up-regulation in high-FE pigs (red, log2FC > 1; pink, 0 < log2FC < 1), while neon green and light green indicate down-regulation in high-FE pigs (neon green, log2FC < −1; light green, −1 < log2FC < 0).

To detect the significant GO terms and KEGG pathways of the DE genes, function enrichment analysis performed by using DAVID Bioinformatics Resources 6.8. Molecular functional GO analysis identified three significantly (*p*-value < 0.05) enriched GO terms, including calcium ion binding, gamma-aminobutyric acid (GABA)-gated chloride ion channel activity, and oxygen binding (Figure 4 and File S3). Moreover, the top GO term, calcium ion binding, contained the highest number of DE genes. The results of the KEGG pathway analysis showed that DE genes were significantly enriched in mineral absorption, malaria and axon guidance (Figure 4 and File S3). *S100G*, S100 Ca-binding protein G, within the top pathway also appeared in the calcium ion binding process. In addition, the results of biological process GO analysis revealed that the positive regulation of cAMP metabolic process was enriched (Figure 4 and File S3). This metabolic process is related to calcium ion binding and lipolysis in the KEGG database. Further analysis showed that genes involved in the lipid metabolic processes were also included in the identified DE genes. These genes included *ELOVL7*, *B4GALT6*, *ABCD2*, *NR4A3*, *C1QTNF3*, and *ABCB4*. On the basis of these results, an integrated pathway diagram was constructed to reflect the changes in the key genes and their related lincRNAs between the two FE groups (Figure 5). In the integrated pathway diagram, down-regulated and up-regulated genes or lincRNAs in high-FE pigs were colored in green or red, respectively.

## 4. Discussion

Given that improving the FE of growing pigs is a major priority in the pork industry, the mechanism that underlies feed conservation into body weight should be analyzed. Identification of DE genes and lincRNAs could improve our understanding of the regulation of FE at the gene expression levels and provide the transcriptional information for molecular breeding in pig industry. Therefore, we analyzed the transcriptome of porcine adipose tissues to identify the essential genes, lincRNAs, and pathways associated with the FE of pigs. Our results showed that the Ca^2+^ binding and cAMP signaling pathway affects the feed efficiency of pigs by involving in lipid metabolism in adipose tissues.

Previous studies showed that multiple tissues were related to the FE of pigs, including muscle, liver, duodenum, hypothalamus, and adipose [3,31,32,33], which indicated that these tissues all contribute to the FE of pigs. Our previous studies, in which have used the same population data, showed that the mitochondrial energy metabolism involved-genes in muscle and Vitamin A metabolism involved-genes in liver were differently expressed between high- and low-FE pigs [21,22]. These studies indicated that the energy metabolism between the high- and low-FE pigs were different. The adipose tissue is one of the important tissues involved in energy metabolism as liver and muscle. However, from our data, the difference of average backfat thicknesses between high- and low-FE pigs was not significant [22]. The similar results were reported in a previous study, after nine generations of selection for RFI, the backfat thickness of pigs was not significantly different between the low RFI line and high RFI line [34]. Thus, we deduce that maybe the difference of adipose tissues between the high- and low-FE pigs is hard to explain by the average backfat thickness. Moreover, the RFI and daily feed intake of the individuals, which used in this study and our previous study, were significantly different between the high- and low-FE pigs [22]. Our results further showed that there were 147 genes and 18 lincRNAs with significantly differential expression in adipose tissues between high and low-FE pigs.

In this study, nine genes enriched in Ca^2+^ binding function were down-regulated in high-FE pigs, but *S100G* was significantly up-regulated. *S100G* encodes calbindin D9k, which is a vitamin D-dependent Ca^2+^ binding protein as an intracellular bumper to buffer Ca^2+^ concentrations [35,36]. Thus, we deduce that the differential expression of genes involved in Ca^2+^ binding may lead to the difference of intracellular Ca^2+^ levels between high- and low-FE pigs. In human adipocytes, increased Ca^2+^ levels can decrease cAMP levels through PDE activation, which consequently decreases HSL phosphorylation and ultimately inhibits lipolysis [9]. In a previous study of the adipose tissue of pigs indicated a significant decline in backfat thickness due to dietary supplementation of fulvic acid related to the increased activity of HSL [37]. We found that *PDE1A* (encoding PDE) and *LIPE* (encoding HSL) levels declined in the high-FE pigs, though the downward trends were not significantly different between two FE groups (Figure 5). However, maybe this is the reason that the average backfat thicknesses of high- and low-FE pigs showed no significant difference. Although the down-regulation of *PDE1A* and *LIPE* were not significant between two groups, genes involved in lipid metabolic processes (*B4GALT6, ELOVL7, NR4A3, ABCD2, C1QTNF3,* and *ABCB4*) were significantly down-regulated in high-FE pigs (Figure 5). Thus, these results further indicated that the lipid metabolism was possibly decreased in the adipose tissues of high-FE pigs. 

In addition, agonists coupled to the G-protein subunit Gs activating adenylate cyclase (*ADCY1* shown in Figure 5) excite the cAMP-mediated PKA pathway in lipolysis induction [12]. The G-protein-coupled receptor subunit can open Ca^2+^ channels either by directly binding to a Ca^2+^ channel or by indirectly stimulating cAMP production by PKA, which is also capable of opening Ca^2+^ channels (*ATP2B2* shown in Figure 5) [38,39]. We found that G-protein-coupled receptor signaling pathway, which involved genes *GPBAR1*, *OPRD1*, *RGS22*, *GPR153*, and *VIPR2*, were significantly down-regulated in the high-FE pigs. For example, *VIPR2*, a transmembrane glycoprotein that couples to G proteins (*GNAS* shown in Figure 5) to stimulate cAMP production [40], was significantly down-regulated in high-FE pigs (Figure 5). In the cAMP signaling pathway, the Ca^2+^ efflux pump (*ATP2B2*) extrudes Ca^2+^ from the cytosol into the extracellular space [41], and the ATPase pump of Na^+^/K^+^ (*ATP1A4*) performs the ATPase-coupled extrusion of three cytoplasmic Na^+^ ions in exchange for the import of two extracellular K^+^ ions [42]. *ATP2B2* and *ATP1A4* were both down-regulated in high-FE pigs (Figure 5). These results imply that high-FE pigs may have weaker cAMP signals than low-FE pigs. Therefore, lipid metabolic process at the downstream of the cAMP signaling pathway may be affected in adipose tissue (Figure 5). However, previous studies showed that the expression of lipid metabolic processes and lipid catabolism processes involved-genes were varied in high- and low-FE pigs [3,5]. After developing pig lines that differed in RFI, Lkhagvadorj found that 311 genes of adipose tissues DE owing to RFI differences and lipid metabolic processes were enriched by down-regulated genes in low-RFI (high-FE) pigs compared to high-RFI (low-FE) pigs [3]. Gondret has reported that lipid catabolism processes are up-regulated in adipose tissues of low-RFI pigs relative to that in adipose tissues of high-RFI pigs [5]. In this study, *ABCD2* and *NR4A3*, which are involved in the positive regulation of fatty acid oxidation [43,44], were down-regulated in high-FE pigs. Moreover, the lipid biosynthetic process-related genes *ELOVL7* and *B4GALT6* [45,46] and lipid homeostasis-related genes *C1QTNF3* and *ABCB4* [47,48] were also down-regulated in high-FE pigs (Figure 5). These results indicated that cAMP and Ca^2+^ are involved in regulating FE by influencing lipid metabolism in adipose tissues of pigs.

Our comparative analysis revealed 18 DE lincRNAs between high- and low-FE pigs. LincRNAs have been reported involving in the regulation of gene expressions and several lincRNAs have been identified in the regulation of lipid metabolism [49,50]. In our previous study, DE lincRNAs of liver tissues between high- and low-FE pigs imply the influence of lincRNAs to FE [21]. In addition, 18 DE lincRNAs were identified in this study, *linc-sscg0063*, *linc-sscg3186*, and *linc-sscg0918* among them were associated with more than one gene, including *B4GALT6*, *ABCD2*, *NR4A3*, *C1QTNF3*, and *VIPR2* (Figure 5). And these DE lincRNAs and genes were positively correlated. Therefore, DE lincRNAs may affect the FE of pigs by being involved in lipid metabolism.

To summarize, our work is the first to use RNA-seq to identify and annotate DE genes and lincRNAs in the adipose tissues of high- and low-FE pigs. Our results provide candidate genes and lincRNAs, which can improve the explanation of complex mechanisms of regulation on FE of pigs at the transcriptional level. In brief, cAMP, a second messenger, affects the FE of pigs through involving in the regulation of lipid metabolism. Probable candidate genes, *VIPR2*, *ABCD2*, *NR4A3*, *S100G*, *ATP2B2,* and *ATP1A4* may have effects on the FE of pigs. Furthermore, DE lincRNAs affects the FE of pigs by association with genes involved in lipid metabolism.

## Figures and Tables

**Table 1:** Summary of RNA-seq data from six adipose samples (*Sus scrofa* 10.2 genome).

Group	Sample	Input Reads	Mapped Reads	Aligned Pairs	Uniquely Aligned Pairs
High	H1	35408600	31227502 (88.19%)	14169507 (80.03%)	13222135 (74.68%)
	H2	33884370	30244478 (89.26%)	13934050 (82.24%)	13073710 (77.17%)
	H3	36202560	31905042 (88.13%)	14659340 (80.99%)	13634859 (75.33%)
Low	L1	39969940	34795576 (87.05%)	16005710 (80.09%)	14889388 (74.50%)
	L2	36467590	32541034 (89.23%)	14934422 (81.91%)	14000346 (76.78%)
	L3	38732934	34441474 (88.92%)	15774510 (81.45%)	14733075 (76.08%)

**Table 2:** Summary of RNA-seq data from six adipose samples (*S. scrofa* 11.1 genome).

Group	Sample	Input Reads	Mapped Reads	Aligned Pairs	Uniquely Aligned Pairs
High	H1	35408600	33379181 (94.27%)	16218722 (91.61%)	15853890 (89.55%)
	H2	33884370	32306327 (95.34%)	15789892 (93.20%)	15485144 (91.40%)
	H3	36202560	34545431 (95.42%)	16884547 (93.28%)	16485731 (91.07%)
Low	L1	39969940	37850914 (94.70%)	18481876 (92.48%)	17752593 (88.83%)
	L2	36467590	34697774 (95.15%)	16935896 (92.88%)	16562230 (90.83%)
	L3	38732934	36654157 (94.63%)	17854715 (92.19%)	16734175 (86.41%)

## Supplementary Materials

Table S1: PCR primers of six selected different expressed transcripts. Figure S1: The expression levels of reference genes in six adipose tissue samples. File S1: Differentially expressed genes and lincRNAs between high- and low-FE pigs. File S2: Correlated lincRNA-gene pairs in the adipose tissues of high- and low-FE pigs. File S3: Results of functional enrichment in GO molecular function, KEGG pathway, and GO biological process.
