# Supplementary material for: Transcriptome Analysis of Adipose Tissue Indicates That the cAMP Signaling Pathway Affects the Feed Efficiency of Pigs
Source: Genes (Basel). 2018 Jul 4;9(7):336. doi: 10.3390/genes9070336 (PMC6070815; doi:10.3390/genes9070336)
Supplement: Supplementary file 1 [file genes-09-00336-s001.zip › Table S1.docx]

Table S1 PCR primers of 6 selected different expressed transcripts

| **Number** | **Symbol** | **Primer sequence 5’-3’** | **Product length** |
| --- | --- | --- | --- |
| ***S100G*** | F | AAGAAGGGGATCCAAACCAGC | 100 bp |
|  | R | AGGTCATCTAGGGTTCTCGGA |  |
| ***VIPR2*** | F | CCCGTGAACAGCATTCATCC | 130 bp |
|  | R | TGATGTTGTCCCAGACACCG |  |
| ***ELOVL7*** | F | CTCCCGTGTCCGGGGATTC | 106 bp |
|  | R | CGCACAGTCCTAGCTGTAAGA |  |
| ***Linc-sscg2463*** | F | GACAGTCCAAGCGTCACAAC | 280 bp |
|  | R | GTGACGCTCTCACTTCACGG |  |
| ***Linc-sscg3186*** | F | CCCTGACATTCAGCGTCTCC | 174 bp |
|  | R | GGCAAACTTGACCTACCGAGA |  |
| ***Linc-sscg0410*** | F | TGCCCTCAAGCCAGTTTCTT | 109 bp |
|  | R | CGGCCAACCATGTCATCCT |  |

F, forward; R, reverse.
