# Supplementary figures and images for: Transcriptome Analysis of Adipose Tissue Indicates That the cAMP Signaling Pathway Affects the Feed Efficiency of Pigs
Source: Genes (Basel). 2018 Jul 4;9(7):336. doi: 10.3390/genes9070336 (PMC6070815; doi:10.3390/genes9070336)

**Figure S1: The expression levels of reference genes in 6 adipose tissue samples.**


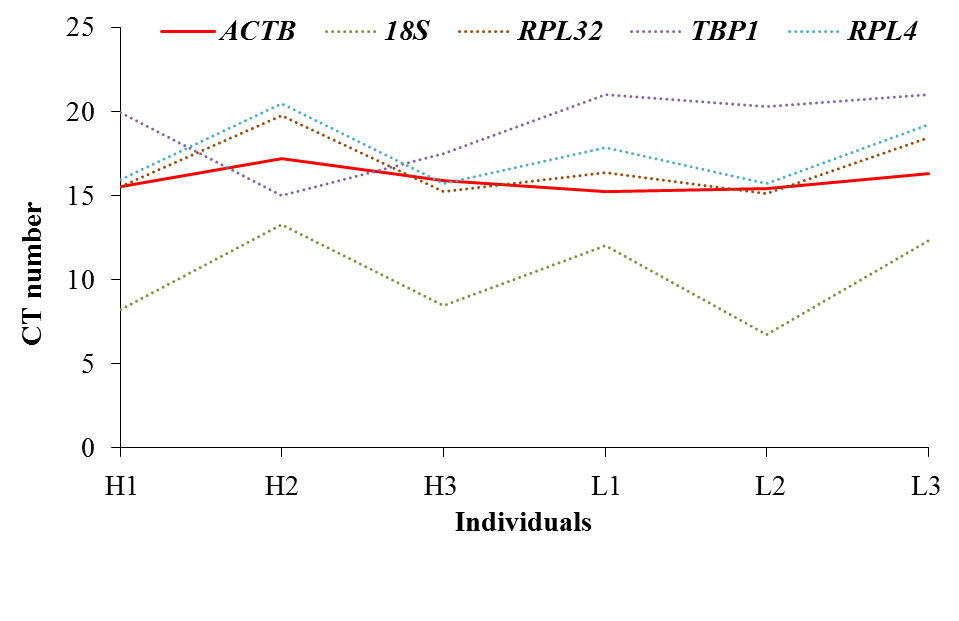


H, high-FE; L, low-FE.

Supplement: Supplementary file 1 [file genes-09-00336-s001.zip › Figure S1.docx]
